# A Dissertation on Some Ancient Plants of Egypt

**Published:** 1832-11

**Authors:** M. Bonastre, G. G. Sigmond

**Affiliations:** Corresponding Member of the Medico-Botanical Society of London.; Secretary.


TRANSACTIONS OF THE MEDICO-BOTANICAL SOCIETY OF LONDON,

*Published under the Direction of the President and Council.*

### ANCIENT PLANTS OF EGYPT.

A DISSERTATION ON SOME ANCIENT PLANTS OF EGYPT. By M. BONASTRE, Corresponding Member of the Medico-Botanical Society of London. Translated from the French by G. G. SIGMOND, M.D., Secretary.

EVERY thing which tends to recall to our recollection the sciences or the arts of ancient Egypt is sure to excite at the present day, in the minds of the most enlightened men, the highest esteem for its former inhabitants.

Since the important labours of Dr. YOUNG upon hieroglyphics, and the learned discovery of M. CHAMPOLLION the younger, upon the interpretation of the hieroglyphic system of ancient Egypt, the history of that country daily acquires a higher degree of interest. There exists, nevertheless, a branch of natural science highly essential, upon which we have but a very imperfect idea, and that part is the botany of ancient Egypt. The Medico-Botanical Society of London having honoured me with the title of corresponding member, I have thought it my duty, as an expression of my gratitude, to transmit a succinct memoir upon some vegetables found in the interior of the coffins of Egyptian mummies of the highest antiquity. I accompany this memoir with some drawings which have been taken from nature; I also send some ancient fruit.

The first is the fruit of the Mimusops, from μιμους and οφις, *monkey face*: Octandria Monogynia: family Saponaceæ of JUSSIEU.

This fruit is often met with in Egyptian tombs, conjointly with the fig Sycamore, enclosed in a little basket variously coloured. The Mimusops Elengi is a proof of the great vicissitudes to which Egypt has been exposed; for this vegetable has entirely disappeared from the soil. No botanical work yet published upon that celebrated country makes mention of the Mimusops Elengi; and I have in vain consulted the Flora of Palestine, by HASELQUIST, that of Egypt by PROSPER ALPINUS, or that of Arabia by FORSKHAL, or the illustration of the Flora of Egypt by DELILE: none of these works indicate that the Elengi now actually exists in Egypt. The Mimusops is only found in the island of Amboine, and some of the isles of the Indian Ocean. Its flowers exhale a most agreeable odour, which gives much pleasure to the females of the country, who perfume their apartments with it.

No. 2. *The Fruit Diospyros Lotus. Polygamia Diœcia Plaqueminier; the Lotus.*

The fruit of the Diospyros does not form part of the Egyptian collection in the museum of the Louvre. It has been recently discovered by M. Passa-Lacqua, and now constitutes a part of the Berlin museum. M. Kunth, a distinguished botanist, has decided that this fruit belongs to the genus Diospyros, a species of lotus, which, I believe, modern botanists refer to the genus Celtis of Theophrastus.

No. 3. *Myrobalan d'Egypte*, of Rauwolf; *Balanites Egyptiaca*, Delille, Fl. Egypte; *Xymenia Egyptiaca*, Desfontaines; *El Eglyg*, of the Arabs of Fazoql; *El Ka*, of the Heathens. *Decandria Monog. Terebintaceæ.*

This ancient fruit was discovered in a little votive basket, which had served to contain offerings to the gods of Egypt, and it is frequently found in the coffins of mummies. This Myrobalan is furnished with a sort of spongy bark, more or less thick; the pellicle which covers it in its state of antiquity is of a red colour, sometimes shadowed with violet.

The stone is marked longitudinally, the sides forming five to six rather saliant angles; the shell is rather thick; the interior of the shell is filled by a kernel of a reddish brown colour, containing a quantity of very fat oil, black, rancid, and excessively acrid. The most marked character of this Myrobalan of Egypt, and which distinguishes it from all the other species, is a kind of spongy circle, placed at the point of insertion of the pedunculus, and which surrounds this organ like a little crown.

My investigation was directed to a very remarkable circumstance, which was, that the little basket that contained the Myrobalan, contained also Myrrha and Bdellium, in large fragments. Is this rencontre the effect of chance? or is it an indirect indication that Myrrha and Bdellium are produced from a vegetable of this genus? And what contributes to add some weight to this supposition is, that several druggists and apothecaries of my acquaintance have frequently brought me the nuts of the Balanistes, which they had found in cases of Bdellium, and which I have also myself collected, in a similar way, among some recently imported.

*My opinion on the Tree that produces the Myrrha and Bdellium.*

Myrrha and bdellium are often found among the substances that served for the process of embalming the ancient Egyptians; and balanistes is found also among them. I have sought to explain the origin of the word Myrobalanus, and I

have found that it was formed from two Greek words, Μυῤῥα, *myrrh*, a perfume, and βάλανος, *fructus-glans*, fruit; as if they had said, fruit or acorn of the tree that produces the myrrh, or perfume.*

THEOPHRASTUS, book ix. chap. iv., informs us that the myrrh tree was certainly thorny: φλοίον ακανθιώδη και όν λεῖον. Now the Balanistes was thorny also.

The opinion of BRUCE, who attributes the myrrh to a species of Mimosa or Acacia, has long been looked upon as an error, from the circumstance, as Dr. DUNCAN very judiciously remarks, in the Edinburgh New Dispensatory, that mimosas furnish but simple gum, and not the gum resin.

EHRENBERG announces that he has found, both in Nubia and Arabia, a shrub from which he has frequently collected myrrh, similar to the myrrh of commerce.

NEES VON ESENBECK has drawn this shrub from the specimens introduced by Ehrenberg, and which present the generic features of Balsamodendrum, or the Amyris of LINNÆUS. Nees calls it the *Balsamodendrum Myrrha.*

But I shall on this subject take the liberty of making an observation similar to that of Dr. Duncan's. The Amyris Opobalsamum, or, as it would be better to call it, the Gilead Balsamodendrum, will be found, on analysis, to produce but pure resin; that is to say, resins or bastard balms, which are perfectly soluble in alcohol and ether, and which do not contain more gum than the produce of the mimosas would resin. Besides, we know, from PLINY and DE THEVENOT, that the trees which produce the myrrh and the bdellium were thorny, and that they grew in the same wood.

I have analysed a new species of myrrh that has been lately imported, and find that it is composed of several principles, as follow:

*Analysis of a new Species of the Myrrh of Commerce.*

| | Parts. |
|---|---|
| Gum, soluble . . . . . . | 50 |
| ——, insoluble . . . . . . | |
| Rosin, soluble, and subresin . | 38 |
| Oil, volatile, fluid . . . . . | 3 |
| A bitter extract, non-resinous . | 4 |
| Acid, not determined | |
| Salt, potass base . . . . . . | 5 |
| ———— chalk . . . . . | |
| Silica, adhering only | |
| | 100 |

* The most part of the other species of the fruit Myrobalanus should have a similar etymology.

This species of myrrh does not differ much in its constituent parts from the ancient myrrh of Troglodytia; but a very important remark, and one first made by myself, is that real myrrh turns red, and even blue, on coming in contact with nitric acid, placed under certain circumstances, which does not happen with the new species.

We are no more acquainted with the tree that produces the myrrh, than we are with the tree that produces the bdellium; but, having been frequently struck with the repeated presence of the nuts of the Balanistes Egyptiaca with myrrh and bdellium in ancient monuments, and especially in some cases of bdellium recently imported into France, I have no difficulty in supposing that these balanistes may furnish one or the other of these two resinous gums. However, I do not lean exclusively to this opinion; and, as the tree is thorny according to Theophrastus, the presence of the fruit of the balanistes may be accounted for by supposing it to have fallen from some neighbouring tree, at the moment the Arabs were collecting the crops of myrrh and of bdellium.

I relate this circumstance to prove to the Medico-Botanical Society of London that I have already occupied myself with the proposed question. I will also add, that on the departure for Egypt, in 1829, of the commission of French savans, I gave the necessary instructions for procuring a specimen of the tree that produces the real myrrh; but, the labours of this commission having been directed to objects of antiquity of quite another nature, I have not been able to obtain the information. On the other hand, the French savans ascended the Nile only as far as the second cataract; but it is in a much higher latitude, in a country much more dangerous to traverse, that the tree grows which produces the real myrrh. I possess on this subject some very circumstantial details.

No. 4 is the *Fruit of the Rhamnus Lotus* of the famed tree of the *Lotophagi.*

The fruit of which, sweet as honey, had on foreigners the effect of banishing the regret they felt for their country. This fruit, as is well known, is a species of the Jujube tree, Ziziphus Lotus, or may be that of Nabeca, which has an extraordinary sweet taste: it is originally from Africa. The nut is hard, and rather of an elongated shape; the kernel has become black, through the lapse of ages; its resemblance is perfect. This species of lotos, λοτοφάγον δενδρον ζ, was found in a small votive basket, full of offerings.

No. 5. *Fruit of the Pine, Pinus Pinea.*

It was discovered, as well as a cone of cedar of Lebanon,

(Cedrus Lebani,) in the catacomb of Thebes. These two fruits form a part of the Egyptian museum at Paris: they are the only two of the species that exist in an antique state.

No. 6, are *Seeds of the Lepdium; λεπιδιον* of Dioscor.

No. 7. *The Grain or Seed of Mimusops Elengi.*

No. 8. *Corn; Triticum Æstivium; πυρας* of Homer.

This corn is a little better preserved, and was discovered in a vase of red clay, which was enclosed in the tomb of an agriculturist.

No. 9, *Barley; Hordeum distichum; κριρή*, (from the same tomb.)

No. 10. *Raisins; Vitis vinifera*, in a high state of preservation.

No. 11. *Remains of a Crown or Garland*, which I shall call "*Demotique*," or *Popular*.

This garland was generally formed of leaves of some plant, the genus of which it is difficult to determine, but it has some relation to Unona Æthiopica. The flowers are those of the flowery capitules of the Mimosa nilotica. This kind of garland encircled often the bodies of certain mummies from head to foot.

No. 12. *Date*, of the *Phœnix Dactylifera.*

No. 13. *Arequier*, or *Fruit of a new Species of the Genus Areca:*

Called Areca Pane Lacquæ by M. Kunth, the botanist: the living original is unknown.

No. 14. *Lentilles de Peluse; Lens Pelusiaca.*

This is the far-famed vegetable which is thought to be a lineal descendant of the lentils, for a plate of which Esau sold his birthright to Jacob. These lentils are of a much smaller species than those at present used in France.

The lentils of Pelusium are at present cultivated in the environs of this Egyptian city, in the neighbourhood of that branch of the Nile called Pelusiad, and from which the species derives its name. This vegetable is extremely difficult to naturalize in France. I send some to Mr. Humphry Gibbs, in order to make the experiment of raising them in England; an experiment, the result of which I request he will communicate to me at a future period.

No. 15. *Another Leguminous Seed.*

This seed, which I lately discovered in a small vase of clay, is extremely rare. I have not been able to determine its species: whether it is a Lathyrus, a Cicercula, or a species of Trigonella, I am ignorant. This seed is smooth, because it is deprived of the pellicle that surrounded it; it is of an iron-red colour.

Such are the objects of antiquity, the collection of which I request the Medico-Botanical Society of London to accept as a mark of my esteem and my gratitude.

I have the honour to be, with the most profound respect, your very obedient, humble servant.

(Signed) BONASTRE.

*Paris.*

---

TRANSACTIONS OF THE MEDICO-BOTANICAL SOCIETY OF LONDON.
*Published under the Direction of the President and Council.*

